# Graph Theoretical Analysis of Structural Neuroimaging in Temporal Lobe Epilepsy with and without Psychosis

**DOI:** 10.1371/journal.pone.0158728

**Published:** 2016-07-06

**Authors:** Daichi Sone, Hiroshi Matsuda, Miho Ota, Norihide Maikusa, Yukio Kimura, Kaoru Sumida, Kota Yokoyama, Etsuko Imabayashi, Masako Watanabe, Yutaka Watanabe, Mitsutoshi Okazaki, Noriko Sato

**Affiliations:** 1 Department of Radiology, National Center of Neurology and Psychiatry, Tokyo, Japan; 2 Department of Neuropsychiatry, Graduate School of Medicine, the University of Tokyo, Tokyo, Japan; 3 Integrative Brain Imaging Center, National Center of Neurology and Psychiatry, Tokyo, Japan; 4 Department of Mental Disorder Research, National Institute of Neuroscience, National Center of Neurology and Psychiatry, Tokyo, Japan; 5 Department of Psychiatry, National Center of Neurology and Psychiatry, Tokyo, Japan; University Of Cambridge, UNITED KINGDOM

## Abstract

**Purpose:**

Psychosis is one of the most important psychiatric comorbidities in temporal lobe epilepsy (TLE), and its pathophysiology still remains unsolved. We aimed to explore the connectivity differences of structural neuroimaging between TLE with and without psychosis using a graph theoretical analysis, which is an emerging mathematical method to investigate network connections in the brain as a small-world system.

**Materials and Methods:**

We recruited 11 TLE patients with unilateral hippocampal sclerosis (HS) presenting psychosis or having a history of psychosis (TLE-P group). As controls, 15 TLE patients with unilateral HS without any history of psychotic episodes were also recruited (TLE-N group). For graph theoretical analysis, the normalized gray matter images of both groups were subjected to Graph Analysis Toolbox (GAT). As secondary analyses, each group was compared to 14 age- and gender-matched healthy subjects.

**Results:**

The hub node locations were found predominantly in the ipsilateral hemisphere in the TLE-N group, and mainly on the contralateral side in the TLE-P group. The TLE-P group showed significantly higher characteristic path length, transitivity, lower global efficiency, and resilience to random or targeted attack than those of the TLE-N group. The regional comparison in betweenness centrality revealed significantly decreased connectivity in the contralateral temporal lobe, ipsilateral middle frontal gyrus, and bilateral postcentral gyri in the TLE-P group. The healthy subjects showed well-balanced nodes/edges distributions, similar metrics to TLE-N group except for higher small-worldness/modularity/assortativity, and various differences of regional betweenness/clustering.

**Conclusion:**

In TLE with psychosis, graph theoretical analysis of structural imaging revealed disrupted connectivity in the contralateral hemisphere. The network metrics suggested that the existence of psychosis can bring vulnerability and decreased efficiency of the whole-brain network. The sharp differences in structural networks between morphologically homogeneous groups are remarkable and may contribute to a better understanding of psychosis in TLE.

## Introduction

Temporal lobe epilepsy (TLE) is the most common focal epilepsy among adults [1], and psychosis is one of the most important psychiatric comorbidities [2]. While around 10–20% of patients with TLE can be expected to develop psychosis [2], many TLE patients do not. No plausible explanation has yet been proposed for these different phenotypes. As for structural neuroimaging, a previous study has reported no significant difference in gray matter between TLE with and without psychosis using voxel-based morphometry [3]. Although some recent studies have reported slight to moderate cortical abnormalities in epilepsy with psychosis [4–6], their etiologies or types of epilepsy were rather heterogeneous and the results were inconsistent.

Graph theoretical analysis is a mathematical method that enables us to analyze connectivity networks from brain imaging or electrophysiology, and is expected to be very useful for the understanding of epilepsy [7]. Additionally, graph theoretical analysis can be applied to structural MR imaging for evaluation of networks, and recently anatomical covariance methods using regional gray matter volumes have increasingly been reported [8, 9]. There already have been a few reports about the differences between findings in unilateral TLE and healthy subjects [10, 11]. We hypothesized that the existence of psychosis in TLE could affect neuronal networks on structural MRI, and that relatively homogeneous patient groups would be desirable for comparison because the influence of local atrophy on graph theoretical analysis has not yet been thoroughly investigated [12]. In the recent classification, TLE with hippocampal sclerosis (HS) was classified as a distinctive constellation [13]. The aim of this study was use a graph theory technique to investigate connectivity differences in structural imaging of TLE and HS patients with and without psychosis.

## Materials and Methods

### Subjects

We recruited 11 consecutive TLE patients with unilateral HS presenting with psychosis or having a history of psychosis (TLE-P group), who were examined at our institute between February 2014 and October 2015. The diagnosis of TLE was based on the presence of simple partial seizures or complex partial seizures consistent with TLE, and focal epileptiform discharge predominantly in temporal areas as observed by conventional scalp electroencephalography. After the diagnosis of TLE, all patients underwent conventional MRI for the visual evaluation of epileptogenic lesions by one experienced neuroradiologist (N.S.) with 25 years of experience. The existence of unilateral HS was diagnosed by the following criteria: (1) ipsilaterally reduced hippocampal volume; (2) increased T2 signal on the hippocampus; and (3) abnormal morphology; i.e., a loss of internal architecture of the stratum radiatum, a thin layer of white matter that separates the dentate nucleus and Ammon’s horn.

The presence or history of psychosis was diagnosed based on the Diagnostic and Statistical Manual of Mental Disorders, 4th edition criteria [14]. In the same period, we also recruited as controls 15 age-, gender-, and laterality-matched TLE patients with unilateral HS without any history of psychotic episodes (TLE-N group). In addition, 14 age- and gender-matched healthy subjects were also investigated for the purpose of reference (Healthy group).

The exclusion criteria for all subjects were as follows: (1) a significant medical history of acute encephalitis, meningitis, severe head trauma, or ischemic encephalopathy; (2) suspicious epileptogenic lesions (e.g., tumor, cortical dysplasia or vascular malformation) on MRI other than ipsilateral HS; (3) epileptic paroxysms in extra-temporal regions on electroencephalography; or (4) severe mood disorders, personality disorders, developmental disorders, or drug/substance-induced psychoses.

The clinical data of the patients, including gender, age, onset age of seizure and psychosis, duration from seizure onset, types of psychosis, and anti-epileptic and anti-psychotic drugs were also investigated. We obtained written informed consent from all participants, and this study was approved by the Institutional Review Board at the National Center of Neurology and Psychiatry Hospital.

### MRI acquisitions, processing, and morphometry

MRI for all participants was performed on a 3.0-tesla MR system with a 32-channel coil (Philips Medical Systems, Best, The Netherlands). The parameters of three-dimensional sagittal T1-weighted magnetization prepared rapid acquisition with gradient echo (MPRAGE) images were as follows: repetition time (TR)/echo time (TE): 7.12 ms/3.4 ms, flip angle: 10°, number of excitations (NEX): 1, 0.6-mm effective slice thickness with no gap, 300 slices, matrix of 260 × 320, 26 × 24 cm field of view (FOV).

We also added a routine MRI examination by the following three protocols. Transverse conventional T1-weighted images: TR/TE 602/8.0 ms, FA 70°, NEX 1, 3.0-mm thickness with a 1.5-mm gap, 34 slices, matrix 256×174, 23×18 cm FOV, acquisition time 3:33 min. Transverse turbo spin echo T2-weighted images: TR/TE 4704/80 ms, FA 90°, NEX 2, 3.0-mm thickness with a 1.5-mm gap, 34 slices, matrix 368×215, 23×18 cm FOV, acquisition time 2:49 min. Coronal fluid-attenuated inversion recovery images: TR/TE 10000/120 ms, inversion time 2450 ms, FA 120°, NEX 2, 3.0-mm thickness with 1.5-mm gap, 34 slices, matrix 272×144, 23×18 cm FOV, acquisition time 3:00 min.

Subsequently, image data of MPRAGE were analyzed using the statistical parametric mapping 8 software program (SPM8; http://www.fil.ion.ucl.ac.uk/spm/) running in MATLAB 2014a (The Mathworks, Natick, MA, USA). We segmented the 3D T1-weighted MPRAGE images into GM, WM, and CSF images by a unified tissue-segmentation procedure after image-intensity nonuniformity correction. These segmented gray- and white-matter images were then spatially normalized to a customized template in standardized anatomic space using the diffeomorphic anatomical registration using the exponentiated lie toolbox [15]. Each image was then modulated by the Jacobean determinants derived from the spatial normalization by DARTEL and spatially smoothed with an 8-mm full-width at half-maximum Gaussian kernel to decrease spatial noise and compensate for the inexactitude of normalization.

To analyze left and right TLE patients together, images of left TLE patients were left-right flipped to make the right hemisphere the ipsilateral hemisphere before the above-mentioned normalization process in both groups. With this procedure, we aimed to evaluate ipsilateral and contralateral differences. To mitigate the possible left/right bias in the analyses, in addition, we randomly flipped the images of healthy subjects in the similar proportions (50%).

After normalization, we applied the created gray-matter images to the ‘flexible factorial’ design for a multi-group analysis in SPM8 with age and gender as nuisance covariates to confirm morphological differences among groups. Correlations that met the following criteria were deemed significant: a height threshold of *p*<0.001 (uncorrected) and an extent threshold of *p*<0.05 (uncorrected).

### Graph theoretical analysis

Graph Analysis Toolbox (GAT) was used for the graph theoretical analysis in this study [16]. GAT is an open-source package that supplies a graphical user interface to facilitate analysis and comparison of structural brain networks. We applied the normalized gray-matter images of both groups of patients to GAT running in MATLAB 2014a with age and gender as nuisance covariates. As secondary analyses, each group of TLE was also compared to the healthy group respectively for the purpose of reference. The available region of interest (ROI) scheme on GAT comprised the 90 cortical and subcortical regions from the Automated Anatomical Labeling template [17].

For construction of the structural correlation network, GAT analyzed all 90 ROIs, and a 90 × 90 association matrix for each group was generated using the Pearson correlation coefficient. The matrices were thresholded at multiple densities (ranged from 0.10 to 0.50 at intervals of 0.02) and converted into binary adjacency maps. Network hub analysis was also performed. GAT then quantified the network hubs based on measures of betweenness centrality (BC).

Then, the following network metrics were calculated: clustering coefficient (*C)*, a measure of the number of edges that exist between its nearest neighbors; characteristic path length (*L)*, the average shortest path length between all pairs of nodes as a measure of network integration; global efficiency *(E*_*glob*_*)*, the exchange of information across the whole network, which is inversely related to the path length; local efficiency *(E*_*loc*_*)*, the inverse of the average shortest path connecting neighbors of nodes; small-worldness *(σ)*, an indicator of small-worldness, which is calculated as [*C*/C_rand_]/[*L*/L_rand_] where C_rand_ and L_rand_ are the mean clustering coefficient and the characteristic path length of 20 random networks; assortativity, a measure of preference for network's nodes to attach to others that are similar; transitivity, a measure of network segregation; and modularity, a measure of the strength of division of a network into modules.

In addition, the network resilience to random failure and to targeted attack was evaluated. Random failure was assessed by randomly removing one node from the network and measuring changes repetitively, whereas a targeted attack was assessed by removing the nodes in rank order of decreasing nodal BC.

As for statistical regional comparison between groups, GAT was used to perform nonparametric permutation tests and assess the regional difference in BC and clustering of connectivity between the two groups for each comparison.

The above analyses were fully automatically; a previous paper reported the details of the processes [16].

### Statistical analysis

GAT: GAT compared the areas under a curve (AUC) of each network measure and resilience of the groups. To test the significance, the actual between-group difference in AUC for each network measure was placed in the corresponding permutation distribution, and the *p*-value was calculated based on its percentile position. GAT also performed a one-tailed non-parametric permutation test (1000 repetitions) for evaluation of the regional differences in BC and clustering between the two groups for each comparison. This permutation testing adopted a shuffled assignment of each permutation group. In each repetition, in fact, the regional data of each participant were randomly reassigned to one of the two groups, and as a result each randomized group had the same number of subjects as the original groups. To correct for multiple comparisons, a false discovery rate *p*<0.05 was deemed significant.

Clinical demographics: The differences in clinical parameters among the three groups were evaluated using one-way ANOVA for age, Pearson's χ2 test for gender and laterality, and unpaired *t*-tests for the other parameters with SPSS software, ver. 23.0 (SPSS Japan, Tokyo).

## Results

### Demographics

Detailed clinical demographics of the participants are shown in Table 1. There were no significant differences among the three groups or between the two groups of TLE in laterality, sex, mean ages at exam and onset, mean disease duration or mean number of anti-epileptic drugs. Only one patient in the TLE-P group had a history of postictal psychosis, whereas the other 10 patients had interictal psychosis including 5 with chronic psychosis and 5 with episodic psychosis. All 10 patients with interictal psychosis took antipsychotics, whereas all 15 patients in the TLE-N group and the patient with postictal psychosis took no antipsychotics.

**Table 1:** Demographics of patients with temporal lobe epilepsy (TLE) with and without psychosis and healthy subjects.

Feature	TLE-N (n = 15)	TLE-P (n = 11)	Healthy (n = 14)	*p*-value
**Gender (no.)**				
Men:women	8:7	5:6	7:7	0.92
**Age at the examination (years)**				
Mean ± SD	48.7 ± 9.6	50.1 ± 8.6	49.6 ± 8.8	0.92
**Laterality (no.)**				
Hippocampal sclerosis side (L:R)	7:8	5:6	N/A	0.95
**Disease duration (years)**				
Mean onset age ± SD	14.2 ± 8.6	18.5 ± 15.2	N/A	0.36
Mean duration of epilepsy ± SD	34.5 ± 11.4	31.5 ± 15.2	N/A	0.58
Mean onset age of psychosis ± SD	N/A	36.0 ± 15.8	N/A	N/A
**Current treatment (no.)**				
Mean number of AEDs ± SD	2.67 ± 0.98	2.18 ± 1.08	N/A	0.24

TLE-N, TLE without psychosis; TLE-P, TLE with psychosis group; AEDs, anti-epileptic drugs; N/A, not available

### Morphological-analysis

**Fig 1:**
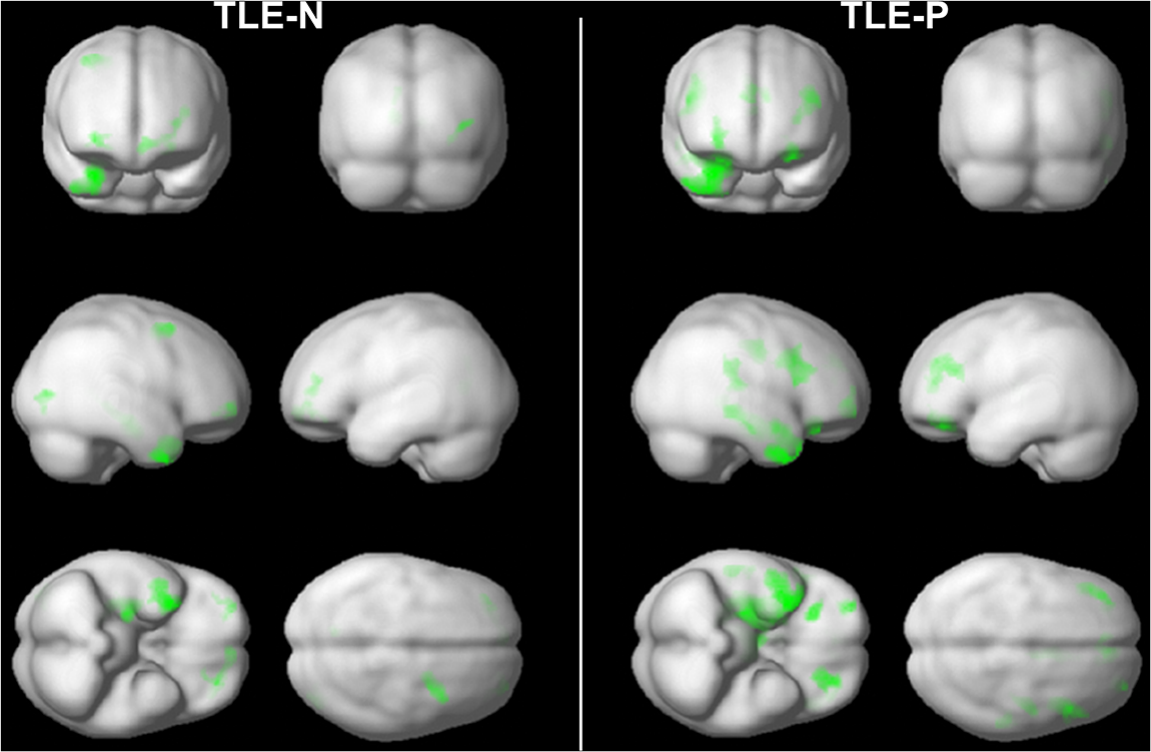
The results of morphological comparison of each TLE group with the healthy subjects. The right side of brain images is the focus side. Colored areas denote significant gray matter reduction: a height threshold of *p*<0.001 (uncorrected) and an extent threshold of *p*<0.05 (uncorrected).

There were no significant differences in morphology between the TLE-N and TLE-P groups. Compared to the healthy subjects, both TLE groups showed various gray matter reductions mainly in the ipsilateral temporal lobe (Fig 1). The TLE-P group appeared to show slightly broader atrophy than the TLE-N group, although the direct comparison was insignificant.

### Graph-analysis

**Fig 2:**
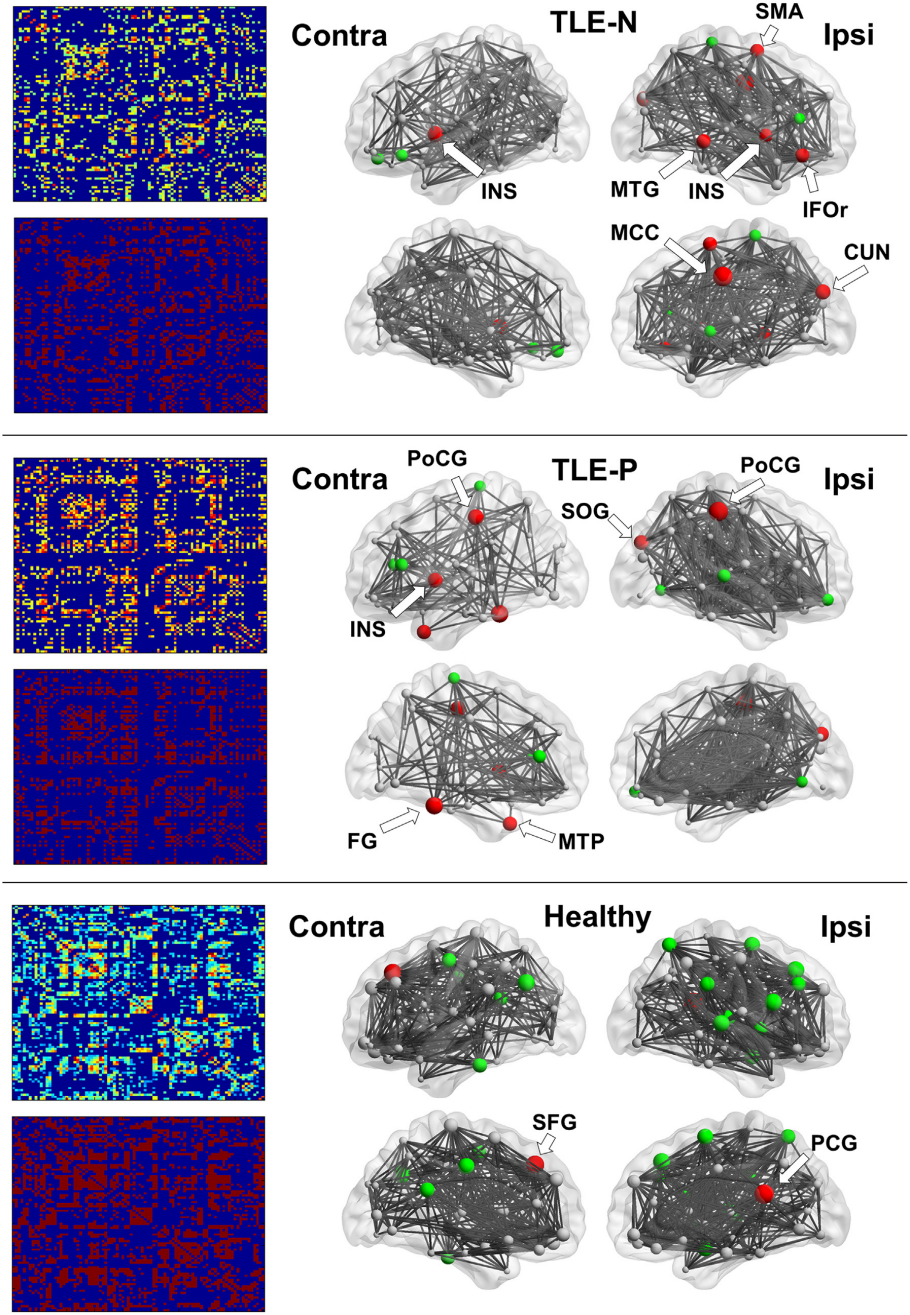
The results of association matrices (left) and network hub nodes and edges (right). The size of nodes indicates the betweenness centrality (BC). Red denotes hub nodes with BC >2SD, and Green denotes those >1SD. CUN, cuneus; FG, fusiform gyrus; IFOr, inferior orbitofrontal cortex; INS, insula; MCC, middle cingulate gyrus; MTG, middle temporal gyrus; MTP, middle temporal pole; PCG, posterior cingulate gyrus; PoCG, postcentral gyrus; SFG, superior frontal gyrus; SMA, supplementary motor area; SOG, superior occipital gyrus. The locations of nodes >1SD are shown in Table 2.

The 90 × 90 association matrices for the three groups are presented in Fig 2. The binary adjacency matrices were then derived by thresholding at the minimal density.

**Fig 3:**
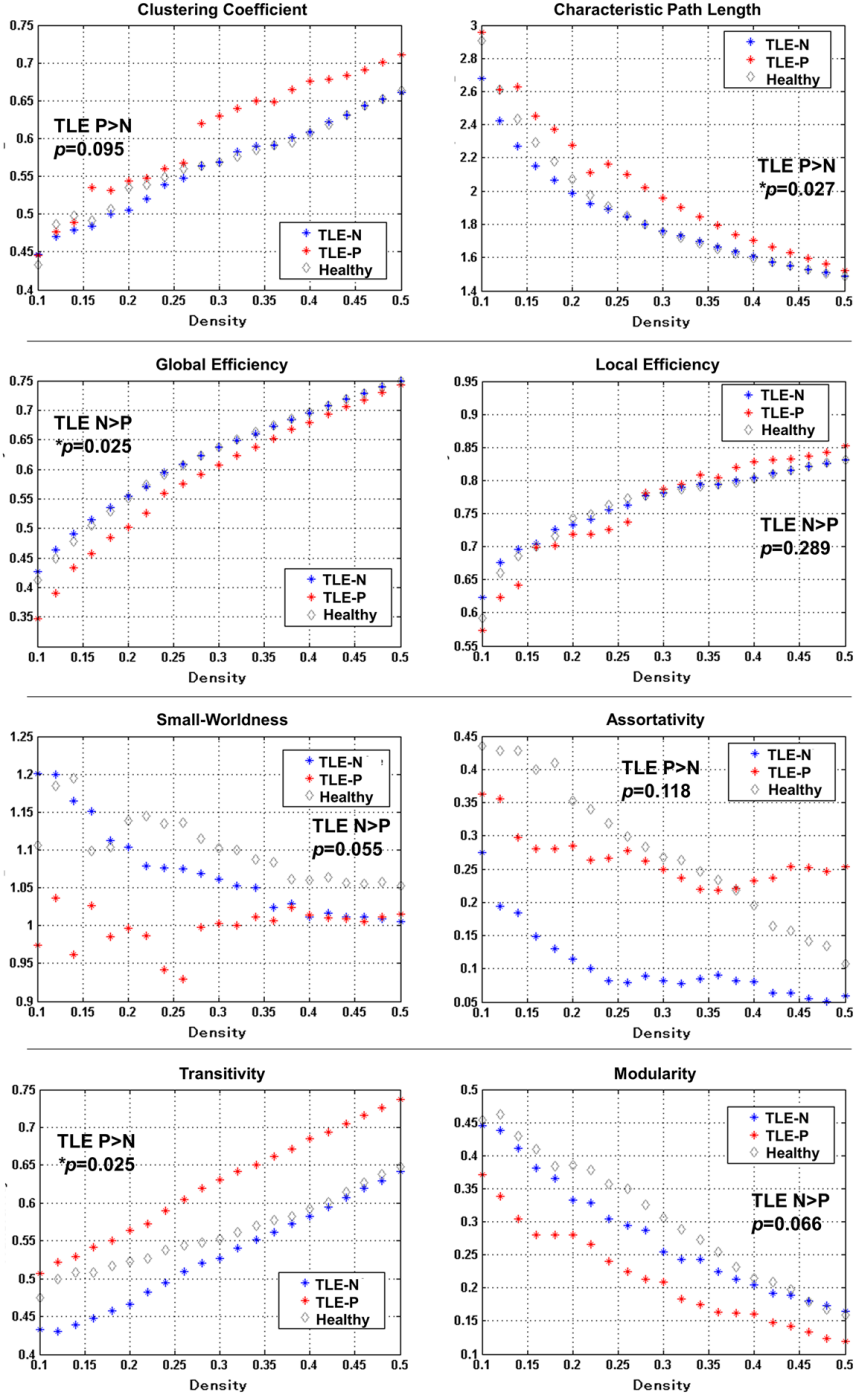
The results of network metrics, and the *p*-values of AUC comparisons between both TLE-N and P groups. The meanings of the measures are described in Methods section of the text.

**Fig 4:**
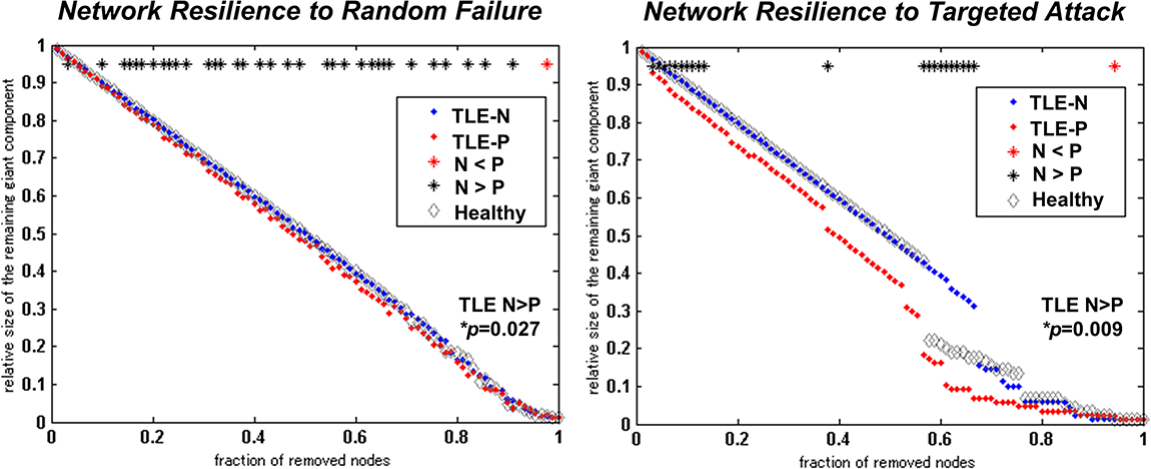
The results of assessments of network resilience to random failure or targeted attack. The *p*-values of AUC comparisons between both TLE-N and P groups are also shown.

The results of network metrics are shown in Fig 3. The TLE-P group showed significantly higher characteristic path length, transitivity, and lower global efficiency than those of the TLE-N group. Additionally, in the TLE-P group, lower small-worldness, modularity, higher clustering coefficient and assortativity were found at a trend level. The results of network resilience to random failure or targeted attack are also presented in Fig 4. The TLE-P group showed significantly lower resilience in both measures. As for the healthy subjects, the metrics and resilience were similar to the TLE-N group except for the higher small-worldness modularity, and assortativity.

Hub regions and edges in the three groups are also presented in Fig 2 and Table 2. In the TLE-P group, reduced connectivity and the number of edges in the contralateral hemisphere were evident, and the contralateral nodes were large in number and size. Hub nodes with a BC value over 2SD were found predominantly in the ipsilateral hemisphere in the TLE-N group, and mainly on the contralateral side in the TLE-P group. The healthy group showed evenly-distributed hubs and edges locations.

**Table 2:** Locations of network hub nodes with betweenness centrality values over 1 or 2SD in the three groups.

	Nodes >2SD	Nodes >1SD
**TLE without psychosis**		
*Ipsilateral side*	Middle cingulate	Inferior frontal, triangular
	Cuneus	Pallidum
	Inferior orbitofrontal	Paracentral lobule
	Insula	
	Supplementary motor	
	Middle temporal	
*Contralateral side*	Insula	Inferior orbitofrontal
		Superior orbitofrontal
**TLE with psychosis**		
*Ipsilateral side*	Postcentral	Lingual
	Superior occipital	Middle frontal
		Superior temporal
*Contralateral side*	Fusiform	Anterior cingulate
	Insula	Inferior frontal, triangular
	Postcentral	Paracentral lobule
	Middle temporal pole	
**Healthy subjects**		
*Ipsilateral side*	Posterior cingulate	Amygdala
		Inferior frontal, operculum
		Middle frontal
		Superior frontal
		Insula
		Superior parietal
		Supplementary motor
		Supramarginal
		Superior temporal
*Contralateral side*	Superior frontal	Angular
		Middle cingulate
		Posterior cingulate
		Heschl
		Precentral
		Inferior temporal

**Fig 5:**
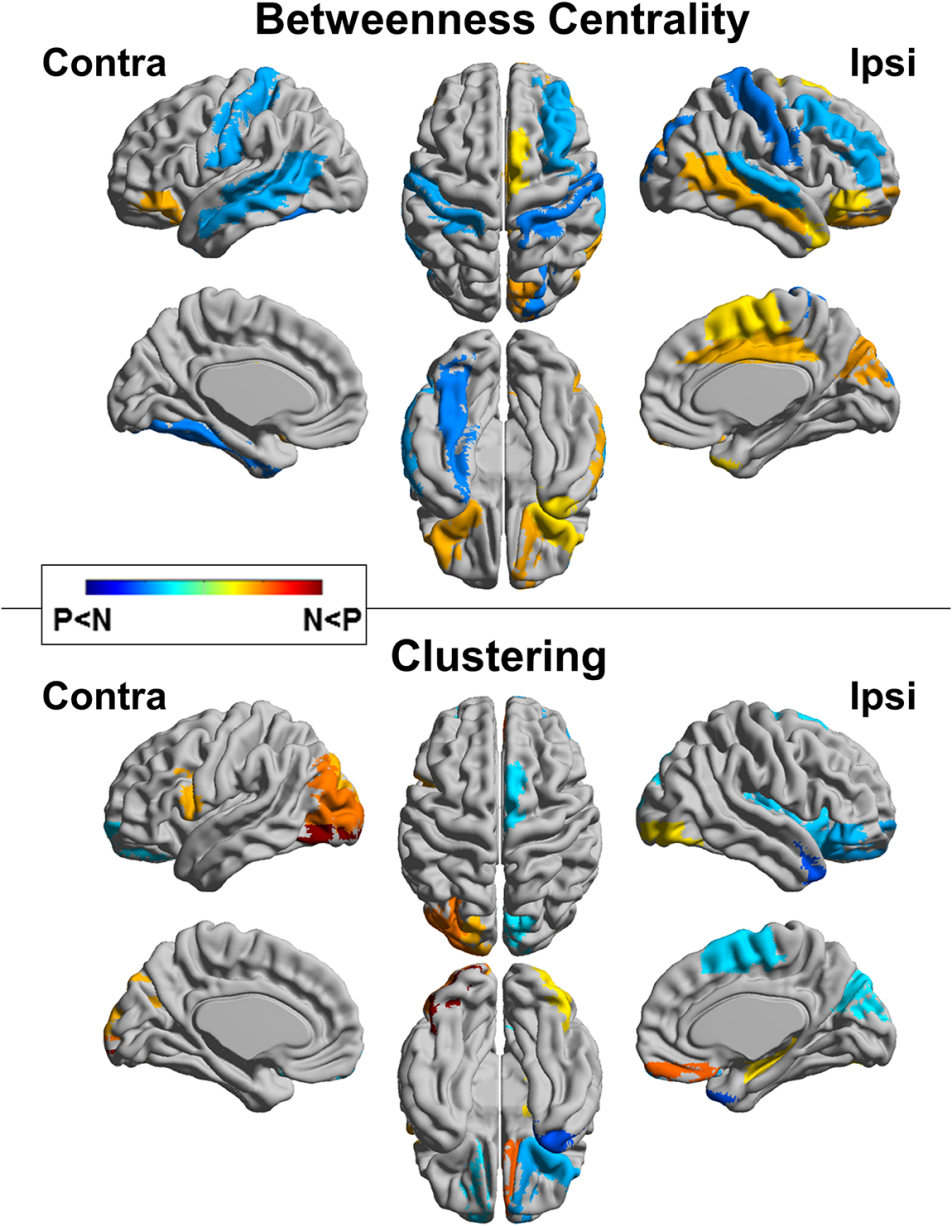
Regional comparisons of the TLE-N and -P groups for betweenness centrality and clustering. Colored areas denote significant differences (a false discovery rate *p*<0.05). N, TLE-N group; P, TLE-P group.

The regional comparison in BC revealed decreased connectivity in the contralateral temporal lobe, ipsilateral middle frontal gyrus, and bilateral postcentral gyri in the TLE-P group. The TLE-P group also showed increased BC in the ipsilateral cingulate gyrus, middle temporal gyrus, and bilateral orbitofrontal areas (Fig 5, upper). The comparison of clustering showed decreased areas in the ipsilateral temporal pole, orbitofrontal cortex, and increased regions in the contralateral occipital lobe in the TLE-P group (Fig 5, lower).

**Fig 6:**
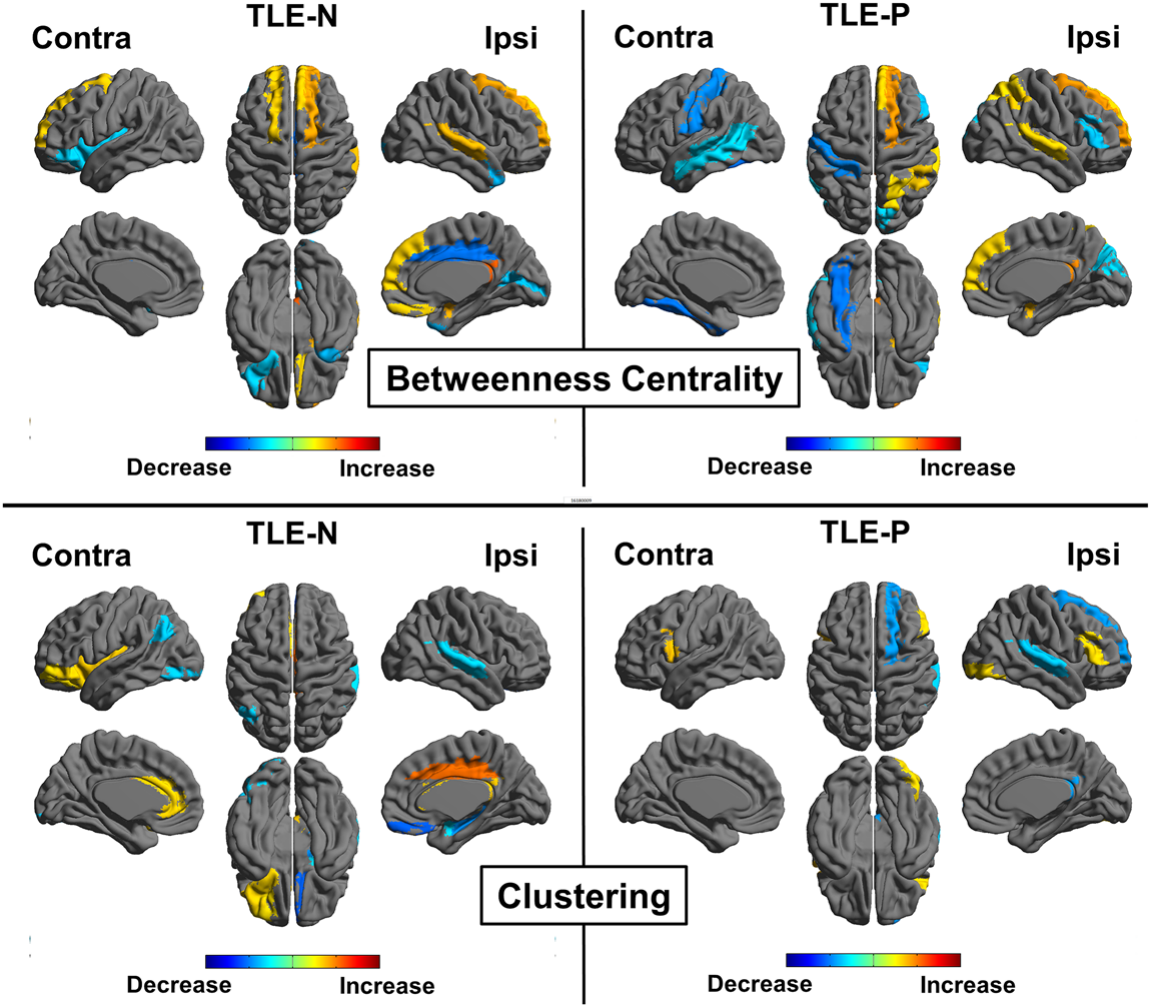
Regional comparisons of each TLE group with the healthy group for betweenness centrality and clustering. Colored areas denote significant differences (a false discovery rate *p*<0.05).

Fig 6 shows the results of comparisons of each TLE group with the healthy subjects. Whereas some common findings were found (e.g. BC increases in superior frontal gyrus), differences were also shown (e.g. decreased BC in the contralateral temporal lobe in the TLE-P group, or increased clustering in the ipsilateral cingulate gyrus in the TLE-N group).

## Discussion

In the present study, we performed graph-theoretical analysis to evaluate connectivity differences on structural imaging of TLE and HS patients with and without psychosis. We also performed additional analyses to compare each TLE group with the healthy subjects. The results suggested that the existence of psychosis in TLE can bring differing hub node distributions, several significant changes of network metrics and resilience, and regional differences in BC and clustering. To the best of our knowledge, this is the first study to evaluate differences relating to the existence of psychosis in TLE with HS using structural imaging and graph theory.

Regarding the hub node and edge distributions, our two TLE groups showed much different patterns. The contralateral number of edges was decreased in TLE with psychosis, possibly suggesting the inflexible function in the contralateral hemisphere. TLE patients without psychosis showed their hub nodes mainly in the ipsilateral hemisphere, which would be consistent with a previous study reporting structural connectivity changes in TLE with graph theory [11]. They reported the normal subjects showed dispersed hub node locations over hemispheres, whereas the hubs of TLE were found primarily in the paralimbic/limbic and temporal association cortices. Especially, the hub nodes in the ipsilateral cuneus, insula, temporal lobe, or orbitofrontal area agreed in part with those of the study. Some other nodes were different, and that might be due to methodological differences such as ROI selection (they used FreeSurfer ROIs) [11]. Additionally the healthy subjects in our study showed well-balanced hub node locations.

In several past studies about partial epilepsies, decreased structural connectivity between the foci and connected areas has been suggested including anatomical covariance methods [18, 19]. In addition, another functional MRI study noted contralateral increased connectivity in unilateral TLE, and proposed a compensatory mechanism [20]. Accordingly, the disrupted contralateral compensatory connectivity can relate to the pathology of psychosis in TLE. Actually, the involvement of the contralateral side in TLE with psychosis has been repeatedly indicated by previous studies with electrophysiological findings [21–23], in apparent accord with our findings. However, there are also some studies reporting increased connectivity of the ipsilateral limbic regions in TLE [24, 25], and then the interpretation of these results should be done in consideration of various differences in modalities or methodologies.

As for the regional comparison, in TLE with psychosis, increased BC was found in the ipsilateral temporal lobe, cingulate gyrus, and bilateral orbitofrontal areas, which are known as limbic association areas [26]. From another view point, TLE without psychosis presented decreased connectivity in these areas, possibly reflecting structural damages by seizure activity. The existence of psychosis might obscure the effects of seizures. The TLE with psychosis group also showed decreased BC in the contralateral temporal lobe, which would relate to the loose connectivity in the contralateral hemisphere as discussed above. Regarding the regional clustering in TLE with psychosis, decreased areas were the ipsilateral temporal pole and bilateral orbitofrontal cortices. Those clustering results appear to be inverse of BC.

We also obtained the results of network metrics in the two TLE groups, and TLE with psychosis showed lower global efficiency, small-worldness and modularity compared to TLE without psychosis. These findings would suggest some dysfunction or vulnerability of the whole-brain networks in TLE with psychosis. Since the metrics of our healthy subjects were similar to those of TLE without psychosis especially in the efficiency metrics, TLE without psychosis would be closer to normal state in efficiency than TLE with psychosis. However, the small-worldness and modularity of TLE without psychosis were not as high as those of the healthy subjects, which could represent the network dysfunction of TLE itself. As for the higher transitivity and assortativity of TLE with psychosis, the results of healthy data were intermediate between both TLE groups, or rather closer to TLE with psychosis, and then the pathological meanings are still unclear. The network resilience to random failure or targeted attack was also decreased in TLE with psychosis with significance. Although our healthy data showed similar resilience with the TLE without psychosis group, possibly due to the small sample size, previous graph theory studies investigating structural connectivity in TLE with larger sample size have already reported decreased global efficiency or deteriorated vulnerability compared to normal controls [10, 11]; the existence of psychosis may introduce further dysfunction to the network efficiency.

Whereas graph theoretical findings in psychosis on TLE are poorly known, there have been a certain amount of studies about those in schizophrenia [27], including those with anatomical covariance methods [28, 29]. In particular, interictal psychosis typically shows schizophrenia-like symptoms such as delusions or hallucinations, whereas postictal psychosis often presents abnormal mood or impaired consciousness [2]. Most of our patients in the group with psychosis had interictal psychosis; thus, our findings might reflect the common pathophysiology between schizophrenia and interictal psychosis.

Network studies about schizophrenia have reported increased path length, clustering coefficient, and decreased global efficiency [30–32], which would be consistent with our findings in TLE with psychosis. In addition, limbic or default-mode network areas including cingulate gyrus or superior frontal cortex were suggested as hub locations or decreased BC regions in schizophrenia [30, 32]. Studies with anatomical covariance methods also reported fronto-temporal network changes [28, 29]. We found regional differences in such areas, but its pathological meanings remain to be elucidated.

There have been several studies about morphological analysis of the cortex in TLE with psychosis [3–6], but the results were heterogeneous. Whereas no cortical abnormality was reported [3], reduced thickness in the inferior frontal gyrus [5], increased thickness in the right anterior cingulate cortex [4] and widespread gray matter reduction [6] were also reported. This heterogeneity could be attributed to the mixing of various etiologies, or mixing of left and right TLE without flipping. In applying structural imaging to graph theoretical analysis, we considered that relatively homogeneous groups would be desirable for comparison, as a previous report showed that any one-sided localized atrophy in the brain was likely to affect the process and results of graph theoretical analyses [12]. In actual fact, we did perform voxel-based morphometry comparison of the two groups using SPM8, and obtained no significant result, although TLE with psychosis showed slightly broader atrophy in comparison with the healthy subjects. Therefore, what is notable in our study is the appearance of very different structural connectivity networks between two morphologically homogeneous groups.

Several studies on functional neuroimaging in TLE with psychosis have reported interesting findings. A study with diffusion tensor imaging suggested microstructural white-matter abnormalities in the bilateral frontal and temporal lobes in TLE with psychosis [33]. Another study reported a tendency for increased blood flow in the right posterior cingulate area in TLE with psychosis [34]. The interpretation of our results in light of these previous findings is somewhat difficult, but further investigations focusing on functional or networking neuroimaging will be important in this field.

This study has several limitations. First, we mainly compare TLE with and without psychosis, but it might be controversial whether the TLE without psychosis group is suitable as controls for evaluation of the effect of psychosis on TLE. Second, we analyzed TLE with psychosis as one group, whereas it contained various types of psychosis such as interictal and postictal psychosis. In addition, we performed a left-right flip procedure in similar proportions of patients in both groups to evaluate ipsilateral and contralateral differences, although this procedure did not allow for comparison between left and right TLE. For investigation of psychiatric disorders, laterality is often important, and then it is unclear whether the altered network configuration is attributable to psychosis (beyond epilepsy). However, the proportion of the laterality in both groups was similar, because we recruited laterality-matched controls. Since the recommended treatment of TLE with HS is early surgical resection [35], and psychosis often occurs with long duration of TLE [36], it was difficult for us to recruit enough cases and controls for subdivided analyses. Furthermore, most of the patients with psychosis took antipsychotic drugs, which also could have affected the results through altered brain function. Our results should be interpreted in light of these limitations.

## Conclusions

In TLE with psychosis, graph theoretical analysis of structural imaging revealed disrupted connectivity in the contralateral hemisphere, and large hub nodes were found mainly on the contralateral side. In addition, the network metrics suggested that the existence of psychosis can bring vulnerability and decreased efficiency of the whole-brain network. The existence of very different structural networks between two morphologically homogeneous groups is remarkable and could contribute to a better understanding of psychosis in TLE.
